# Thermal Deformation Behavior and Dynamic Softening Mechanisms of Zn-2.0Cu-0.15Ti Alloy: An Investigation of Hot Processing Conditions and Flow Stress Behavior

**DOI:** 10.3390/ma16124431

**Published:** 2023-06-16

**Authors:** Guilan Xie, Zhihao Kuang, Jingxin Li, Yating Zhang, Shilei Han, Chengbo Li, Daibo Zhu, Yang Liu

**Affiliations:** 1 School of Mechanical Engineering and Mechanics, Xiangtan University, Xiangtan 411105, China; xieguilan@xtu.edu.cn (G.X.); 17373241475@163.com (Z.K.); 202005501510@smail.xtu.edu.cn (J.L.); 202005501530@smail.xtu.edu.cn (Y.Z.); 16673234565@163.com (S.H.); csulicb@163.com (C.L.); 2 School of Materials Science and Engineering, Nanyang Technological University, Singapore 639798, Singapore; 3 Zhuzhou Smelter Group Co., Ltd., Zhuzhou 412005, China

**Keywords:** Zn-Cu-Ti alloy, hot compression, dynamic material model (DMM), flow stress behavior, softening mechanism

## Abstract

Through isothermal hot compression experiments at various strain rates and temperatures, the thermal deformation behavior of Zn-2.0Cu-0.15Ti alloy is investigated. The Arrhenius-type model is utilized to forecast flow stress behavior. Results show that the Arrhenius-type model accurately reflects the flow behavior in the entire processing region. The dynamic material model (DMM) reveals that the optimal processing region for the hot processing of Zn-2.0Cu-0.15Ti alloy has a maximum efficiency of about 35%, in the temperatures range (493–543 K) and a strain rate range (0.01–0.1 s^−1^). Microstructure analysis demonstrates that the primary dynamic softening mechanism of Zn-2.0Cu-0.15Ti alloy after hot compression is significantly influenced by temperature and strain rate. At low temperature (423 K) and low strain rate (0.1 s^−1^), the interaction of dislocations is the primary mechanism for the softening Zn-2.0Cu-0.15Ti alloys. At a strain rate of 1 s^−1^, the primary mechanism changes to continuous dynamic recrystallization (CDRX). Discontinuous dynamic recrystallization (DDRX) occurs when Zn-2.0Cu-0.15Ti alloy is deformed under the conditions of 523 K/0.1 s^−1^, while twinning dynamic recrystallization (TDRX) and CDRX are observed when the strain rate is 10 s^−1^.

## 1. Introduction

Zinc-based alloys are highly sought after due to their exceptional mix of qualities, such as excellent ductility, weldability, outstanding corrosion resistance [1], and surface aspect, making them suitable for various applications in the medical implantation, building industry, and anti-corrosive fields [2,3,4]. Moreover, these alloys possess strong creep resistance [5], which further increases their demand. The favorable hot workability of these high-strength alloys also contributes to their popularity. Hot extrusion/rolling is the main processing technique used for zinc-based alloys. However, the thermal deformation behavior of the alloys can be affected by several factors [6,7], including temperature, strain rates, and strain [8]. Thus, it is difficult to achieve the parameter specification of zinc-based alloys [9,10,11]. Up to now, the thermal deformation behavior of Zn-Cu-Ti has not been studied in depth. Little consideration has been given to the flow stress evolution and constitutive relationship of the Zn-Cu-Ti alloy during hot deformation.

The constitutive equations and the processing maps are important methods to study the hot workability characteristics and the deformation mechanisms of zinc-based alloys [12,13]. Unfortunately, the traditional Arrhenius equations and 2D processing maps cannot reflect the influence of strain, which has an obvious impact on flow stress [14]. In order to take the strain effect into account, modified Arrhenius equations are used to improve the accuracy of the numerical simulation, overcoming the disadvantages of being time-consuming and labor-intensive [15]. Meanwhile, it can also provide theoretical guidance for the optimization and the predictions of numerical simulations. The 3D processing map established by the dynamic material model (DMM) can reveal the reasonable processing region to optimize the thermal working process [16,17] from which stable and unstable regions can be found. Stable regions are accompanied by dynamic recrystallization [18] (DRX) and dynamic recovery (DRV), which can uniform microstructures and improve processability [19]. However, few systematic studies have revealed the evolution of the microstructure of Zn-Cu-Ti alloys and optimized its forming parameters by 3D processing maps. Therefore, it is of great significance to study flow stress behavior, constitutive equation, processing maps (3D), and dynamic recrystallization (DRX) behavior of Zn-Cu-Ti alloys during hot compression deformation.

The aim of this work is to investigate the thermal deformation behavior of Zn-2.0Cu-0.15Ti alloy under various strains, strain rates, and deformation temperatures. The 3D processing maps of Zn-2.0Cu-0.15Ti alloy are created to optimize the hot deformation parameters and find out the unstable deformation factors. The effects of thermal deformation parameters on the DRX mechanism are analyzed by the electron backscatter diffraction pattern (EBSD) and transmission electron microscopy (TEM), which provides scientific guidance for the thermal deformation behavior and dynamic softening mechanism of Zn-Cu-Ti alloy.

## 2. Experimental Procedure

**Figure 1:**
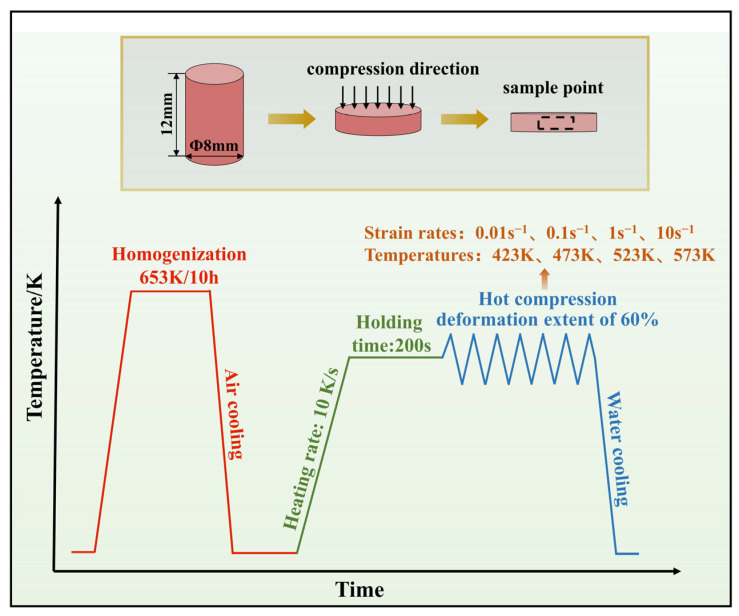
Schematic diagram of hot compression experiment process.

Table 1 lists the component (in wt.%) of Zn-Cu-Ti alloys. In our experiments, using pure Zn (purity 99.995%), high-purity copper foil (99.99%), and high-purity titanium tablet as starting materials for casting samples with a composition of Zn-2.0Cu-0.15Ti (wt.% hereafter). The raw ingredients were melted at 973 K with the protection of argon (Ar) gas in a graphite crucible. The alloy was poured (pouring temperature: 823 K) into the steel mold preheated to 493 K. After natural cooling and solidification, an ingot was obtained. The ingot underwent homogenization at 653 K for 10 h and was used to prepare all the samples. The shape of each specimen for the compression testing is a cylinder (diameter: 8 mm, height: 12 mm). The samples were heated to the specified temperature (423, 473, 523, and 573 K) by using a Gleeble-3500 thermomechanical simulator (Dynamic Systems Inc. America) with a heating rate of 10 K/s. The other experimental parameters were as follows: the strain rates were 0.01 s^−1^, 0.1 s^−1^, 1 s^−1^, and 10 s^−1^, and the height of each sample was compressed by 60%. All compression tests were carried out in a vacuum, and in order to achieve a homogeneous temperature ahead of deformation, all samples were maintained at the necessary temperature for 200 s. Additionally, after the compression test, each sample was promptly water-quenched so that the microstructures could be preserved. Figure 1 depicts the hot compression experimentation process.

Samples for electron backscatter diffraction pattern (EBSD) analysis were prepared into round pieces with a thickness of 100 μm and a diameter of 3 mm. The round pieces were mechanically and vibrationally polished for 0.5 h using a Buehler VibroMet2 Vibratory Polisher (ITW, America), and then the Angle was reduced using a plasma thinning instrument. SEM with a 200 Sirion field emission gun was used for the EBSD studies. OIM7.3 software was used to analyze the EBSD data. Transmission electron microscope (TEM) images were collected using a Tecnai G220 transmission (FEI, America) electron microscope at 200 kV. The shape of TEM samples was similar to EBSD samples, and twin-jet thinning was applied in a mixed solution of HNO3:CH3OH = 1:3. The experimental parameters were 30 V and −243 K, respectively.

## 3. Results and Discussion

### 3.1. Analysis of Flow Stress Curves

**Figure 2:**
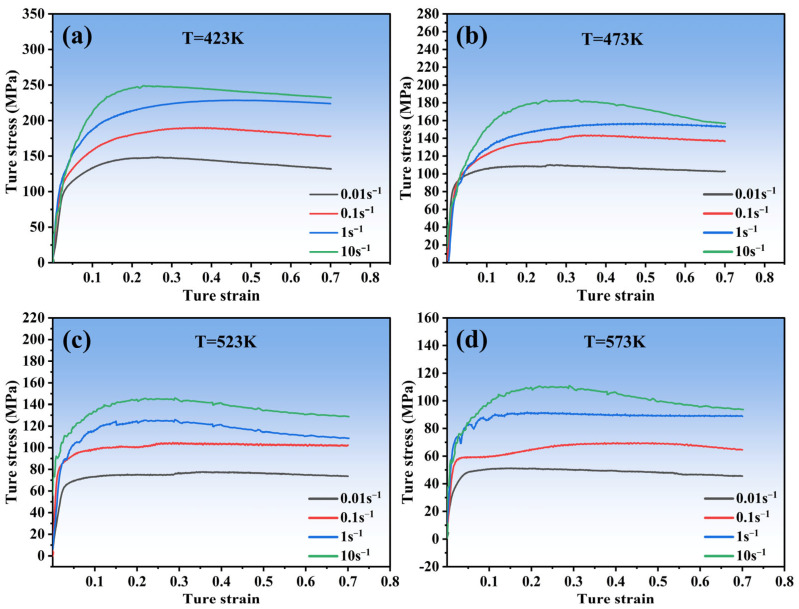
The true stress–strain curves in the hot compression tests of Zn-2.0Cu-0.15Ti alloy at 0.01–10 s^−1^ with a deformation temperature of (**a**) 423 K, (**b**) 473 K, (**c**) 523 K, (**d**) 573 K.

Figure 2 displays the true stress–strain curves of the Zn-2.0Cu-0.15Ti alloy obtained from the compression test conducted at strain rates 0.01, 0.1, 1, and 10 s^−1^ and deformation temperatures 423, 473, 523, and 573 K. The evolution of flow stress curves is roughly divided into three stages. In the first stage, flow stress increases with increasing strain resulting from an increase in dislocation density (work hardening). In the second stage, the increasing rate of flow stress slows down and reaches its peak value, indicating the occurrence of dynamic recovery (DRV) and DRX. In the third stage, flow stress gradually drops until reaching the final steady value. This phenomenon in hot working is usually caused by dynamic recrystallization (DRX) [20,21].

As shown in Figure 2, on the one hand, it can be seen that flow stress decreases with the increase in deformation temperature at a given strain rate. This is because higher temperatures can promote the occurrence of DRX, which causes a decrease in dislocation density. On the other hand, flow stress increases with the increase in strain rates at a given temperature. This is due to the fact that with the limited time for dislocation annihilation to manifest, the dislocation density gradually rises.

### 3.2. Development of Constitutive Equation

The flow stress caused by deformation conditions can be evaluated using the Arrhenius equations, which are presented in Equations (1) and (2) and are commonly utilized for describing thermal deformation behavior [22,23].

(1) $$\dot{\varepsilon }=AF(\sigma )\exp\left(\frac{-Q}{RT}\right)$$

(2) $$F(\sigma )=\{\begin{array}{ll} \sigma^{n_{1}}, & \alpha \sigma <0.8\\ \exp(\beta \sigma ), & \alpha \sigma >1.2\\ \sinh{\left(\alpha \sigma \right)}^{n} & \mathrm{for}\;\mathrm{all}\;\sigma \end{array}$$

For a certain strain, the true stress (MPa) represented by σ, where the strain rate (s^−1^) is represented by $\dot{\varepsilon }$, is calculated by some material constants, including *A*, *n*_1_, *n, α*, and *β*, where *α* = *β*/*n*_1_, as well as gas constant *R* (8.314 J mol^−1^K^−1^), the activation energy *Q* (kJ mol^−1^), and absolute temperature *T* (K).

Meanwhile, combining Equations (1) and (2) leads to the Zener–Hollomon (*Z*) parameter [17,24]:

(3) $$\dot{\varepsilon }=A{\left[\sinh(\alpha \sigma )\right]}^{n}\exp\left(-\frac{Q}{RT}\right)$$

(4) $$Z=\dot{\varepsilon }\exp\left(\frac{Q}{RT}\right)=A{\left[\sinh(\alpha \sigma )\right]}^{n}$$

By substituting Equation (2) into Equation (1) and taking the natural logarithm of both sides, the following equation was obtained:

(5) $$\ln\sigma =\frac{1}{n_{1}}\ln\dot{\varepsilon }-\frac{1}{n_{1}}\ln A_{1}+\frac{Q}{n_{1}RT}$$

(6) $$\sigma =\frac{1}{\beta }\ln\dot{\varepsilon }-\frac{1}{\beta }\ln A_{2}+\frac{Q}{\beta RT}$$

**Figure 3:**
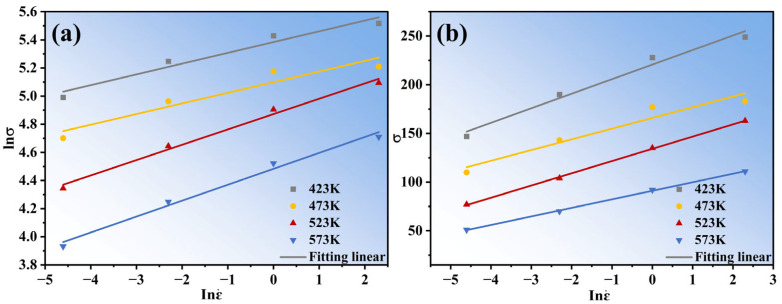
Linear relationship of (**a**) lnσ-ln$\dot{\varepsilon }$ and (**b**) σ-ln$\dot{\varepsilon }$ at different temperatures.

Assuming the material’s activation energy for the deformation is a fixed value unaffected by temperature, according to the true stress–strain curve, the peak stress corresponding to the alloy under various deformation circumstances is determined, and then the ln*σ*-ln$\dot{\varepsilon }$ and *σ*-ln$\dot{\varepsilon }$ curves (Figure 3) can be plotted by linear regression processing according to Equations (5) and (6). Here, the average slopes of the ln*σ*-ln$\dot{\varepsilon }$ and *σ*-ln$\dot{\varepsilon }$ curves are shown by *n*_1_ and *β*, respectively, *n*_1_ = 10.601 and *β* = 0.086 MPa^−1^ can be obtained, so that *α* = *β*/*n*_1_ = 0.007 MPa^−1^.

Equations (7) and (8) are presented using the partial derivative method applied to the logarithm of Equation (3) provided:

(7) $$n=\frac{\partial \ln\dot{\varepsilon }}{\partial [\sinh(\alpha \sigma )]}$$

(8) $$\frac{Q}{\mathrm{Rn}}=\frac{\partial \ln[\sinh(\alpha \sigma )]}{\partial (1/T)}$$

**Figure 4:**
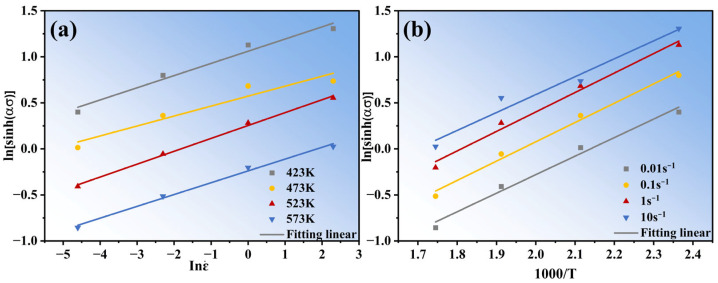
(**a**) Relationships between ln [sinh(ασ)] and ln$\dot{\varepsilon }$ at different temperatures; (**b**) Relationships between ln [sinh(ασ)] and 1000/T at different strain rates.

The value of both parameters ($\sigma ,\dot{\varepsilon }$) is used for the plots of ln [sinh(*ασ*)]-ln$\dot{\varepsilon }$ and ln [sinh(*ασ*)]-1000/T (Figure 4) by linear regression processing according to Equations (7) and (8). By using the linear fitting method, the average values of n and b are calculated to be 7.70001 and 2.534, respectively. Q is calculated to be 130.69 kJ mol^−1^. A is calculated to be 3.33 × 10^12^ s^−1^ by substituting the corresponding data into Equation (5).

Then, the relationship of σ-Z can be defined by Equation (9), and the formula can be written as:

(9) $$\sigma =\frac{1}{\alpha }\ln\left\{{\left(\frac{Z}{A}\right)}^{\frac{1}{n}}+{\left[{\left(\frac{Z}{A}\right)}^{\frac{2}{n}}+1\right]}^{\frac{1}{2}}\right\}$$

The effect of strain on thermal deformation behavior is disregarded based on the aforementioned constitutive equations. Nevertheless, it can be found from Figure 2 that the strain played a considerable role in the true stress. This means that taking the impact of strain into account on the constitutive equations is essential.

**Figure 5:**
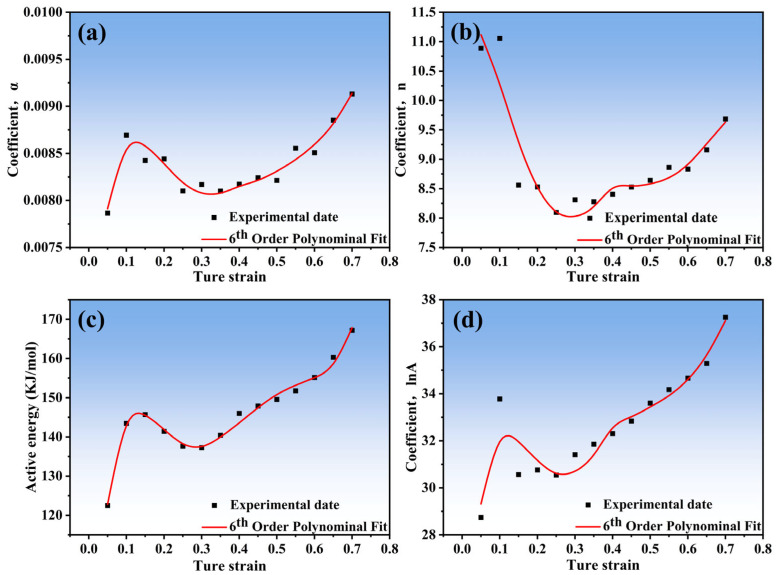
Relationship between the fitted parameters and the strain: (**a**) *a*; (**b**) *n*; (**c**) *Q*; (**d**) ln*A*.

The material constants (*A*, *n*, *Q*, and *a*) are known to be functions of the strain and can be described using polynomial functions [25,26]. To obtain the values of these constants, the strain is varied from 0.05 to 0.7 in increments of 0.05, and the material constants are computed at each strain. The relationship between the material constants and strain is then established through polynomial fitting techniques, as shown in Figure 5. It was found that a six-order polynomial function can accurately depict the impact of strain on material constants, as shown in Equation (10). Figure 5 summarizes the relationship between the material constants and strain. The validity of the seventh-order polynomial model is confirmed by the results of polynomial fitting, which are presented in Table 2.

(10) $$\begin{array}{l} a=h_{0}+h_{1}\varepsilon +h_{2}\varepsilon^{2}+h_{3}\varepsilon^{3}+h_{4}\varepsilon^{4}+h_{5}\varepsilon^{5}+h_{6}\varepsilon^{6}\\ n=i_{0}+i_{1}\varepsilon +i_{2}\varepsilon^{2}+i_{3}\varepsilon^{3}+i_{4}\varepsilon^{4}+i_{5}\varepsilon^{5}+i_{6}\varepsilon^{6}\\ Q=j_{0}+j_{1}\varepsilon +j_{2}\varepsilon^{2}+j_{3}\varepsilon^{3}+j_{4}\varepsilon^{4}+j_{5}\varepsilon^{5}+j_{6}\varepsilon^{6}\\ \ln A=k_{0}+k_{1}\varepsilon +k_{2}\varepsilon^{2}+k_{3}\varepsilon^{3}+k_{4}\varepsilon^{4}+k_{5}\varepsilon^{5}+k_{6}\varepsilon^{6} \end{array}$$

### 3.3. Performance Evaluations

**Figure 6:**
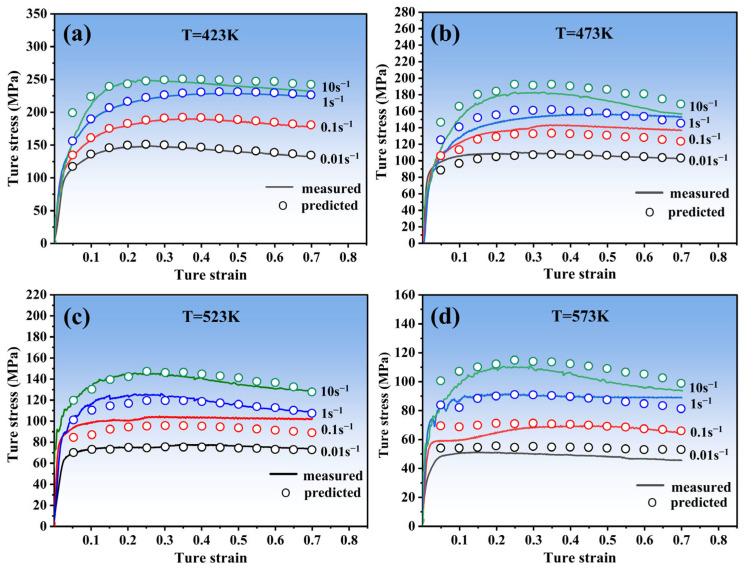
Comparisons between the measured and the predicted true stress at different temperatures: (**a**) 423 K; (**b**) 473 K; (**c**) 523 K; and (**d**) 573 K.

Figure 6 displays that the Arrhenius-type model has good predictability of the flow behavior of Zn-2.0Cu-0.15Ti alloy under various deformation conditions. However, to ensure the reliability of the predictions of the Arrhenius-type model in our work, the mean absolute percentage error (MAPE), correlation coefficient (*R*), and relative error parameters are used to evaluate the reliability. The formula can be written as:

(11) $$R=\frac{\sum_{i=1}^{n}\left(A_{i}-\bar{A}\right)\left(B_{i}-\bar{B}\right)}{\sqrt{\sum_{i=1}^{n}{\left(A_{i}-\bar{A}\right)}^{2}\sum_{i=1}^{n}{\left(B_{i}-\bar{B}\right)}^{2}}}$$

(12) $$\mathrm{MAPE}(\%)=\frac{1}{n}\sum_{i=1}^{n}\left|\frac{A_{i}-B_{i}}{A_{i}}\right|\times 100$$

where n is all the data; *A_i_* and *B_i_* indicate the measured data and the predicted data, respectively. $\bar{A}$ and $\bar{B}$ represent the average values of *A_i_* and *B_i_*, respectively.

**Figure 7:**
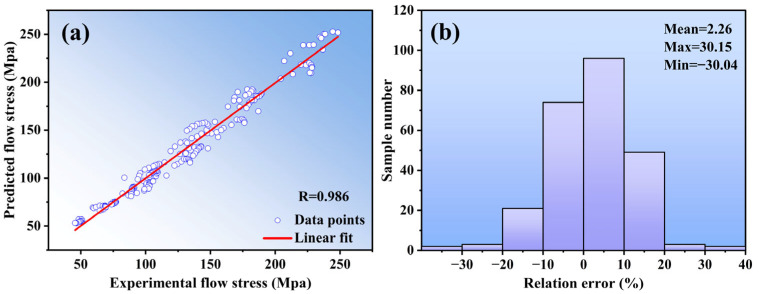
(**a**) Correlation between the experimental and predicted flow stress values obtained from Arrhenius-Type constitutive model; (**b**) Statistical analysis of the relative error by Arrhenius-Type constitutive model.

Generally, *R* is used to reflect the closeness of the correlation between variables [27]. In our study, the closer the *R* is to one, the more accurate the prediction of the model. Furthermore, the MAPE is typically used to reflect the practical prediction errors. The smaller the value of MAPE, the better performance for the predicted model. It can be observed from Figure 7a that *R* was calculated at 0.986. Moreover, the MAPE was 6.48%, and the relative error percentage was used to investigate the predictability performance of the model. One can therefore obtain the following:

(13) $$\mathrm{Relative error}=\left(\frac{A_{i}-B_{i}}{A_{i}}\right)\times 100\%$$

From Figure 7b, the value of the relative error parameter ranges from 30.04 to 30.15%. Combining the above calculation parameters, the result shows that predicted and measured values of the Arrhenius-type model have good agreement in our study.

### 3.4. Processing Maps

At present, Prased proposed hot processing maps by studying the theory of DMM [28,29] and continuum mechanics. In general, the hot processing maps can be obtained by combining the power dissipation map and the instability map. Additionally, in accordance with the theory of DMM, the total power (*P*) of the input system is made up of two components: dissipated co-content *J* and dissipated content *G* [20], which are produced by, respectively, structural change and plastic deformation. The equation appears to be the following:

(14) $$P=\sigma \dot{\varepsilon }=G+J=\int \begin{array}{c} \dot{\varepsilon }\\ 0 \end{array}\sigma d\dot{\varepsilon }+\int \begin{array}{c} \dot{\varepsilon }\\ 0 \end{array}\dot{\varepsilon }d\sigma$$

where *σ* and $\dot{\varepsilon }$ represent the flow stress and the strain rate, the sensitivity of strain rate (m) can be expressed using the following equation:

(15) $$m=\frac{dJ}{dG}=\frac{\partial \ln\sigma }{\partial \ln\dot{\varepsilon }}$$

Typically, the power dissipation efficiency (*η*) is used to assess an alloy’s capacity for power dissipation, which is as follows [30]:

(16) $$\eta =\frac{J}{J_{max}}=\frac{2m}{m+1}$$

When m ≤ 0, there is no power dissipation in the entire system. When 0 < m < 1, it is considered to be in a steady-state flow regime. When m = 1, the value of J is equal to its peak value (*J_max_*). A 3D contour map of power dissipation can express the changes in *η* under various deformation temperatures, strain, and strain rates. Generally, the higher the power consumption efficiency is, the better the performance of alloy processing can be [31], which demonstrates that DRX may occur. However, processes such as adiabatic shear banding and crack growth usually result in structural instability. According to the extreme principle of irreversible thermodynamics proposed by Ziegler [32], the following is an expression for the unstable criterion:

(17) $$\xi (\dot{\varepsilon })=\frac{\partial \ln(\frac{m}{m+1})}{\partial \ln\dot{\varepsilon }}+m<0$$

**Figure 8:**
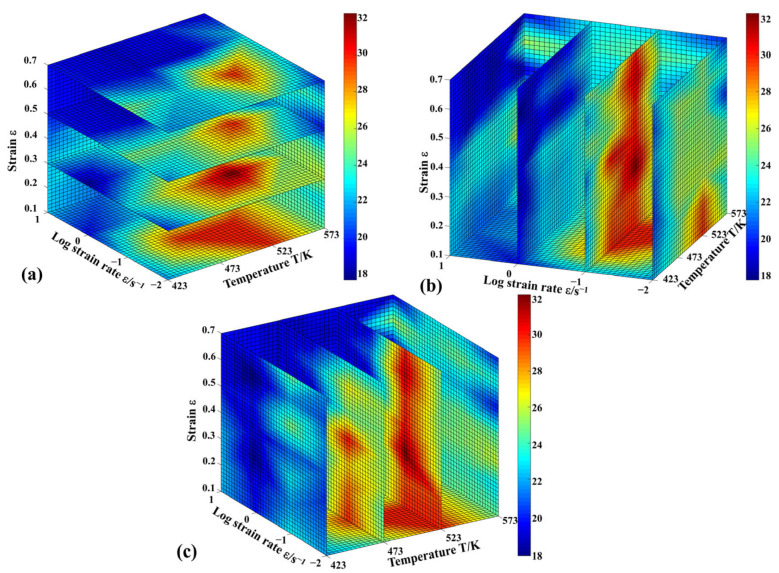
The 3D power dissipation maps of Zn-2.0Cu-0.15Ti alloy: (**a**) strain section, (**b**) strain rate section, and (**c**) temperature section.

**Figure 9:**
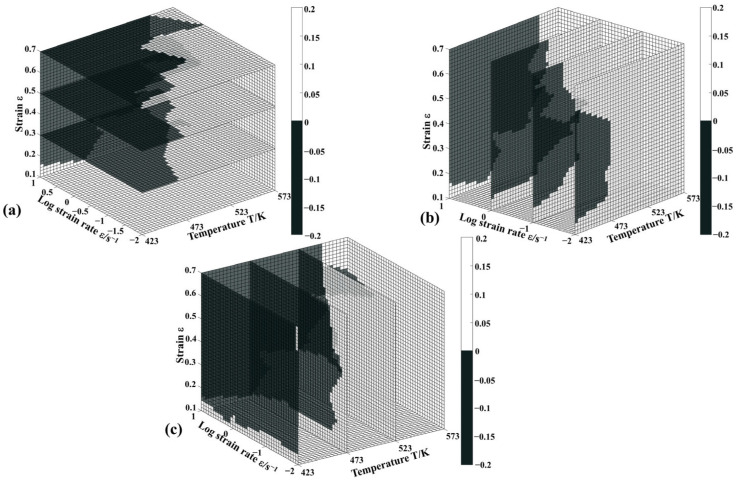
The 3D flow-instability maps of Zn-2.0Cu-0.15Ti alloy: (**a**) strain section, (**b**) strain rate section, and (**c**) temperature section.

The flow behavior of the material can become unstable when the values of the parameters (ξ) mentioned above fall below zero. Figure 8 and Figure 9 illustrate, respectively, the 3D maps of power dissipation and flow instability for the strain range (0.1–0.7), temperature range (423–573 K), and strain rate range (0.01–10 s^−1^). The colored grids of the power dissipation map represent *η*, and the shaded regions of the flow instability map can be used to identify the unstable area. The results suggest that the power dissipation efficiency (*η*) and the flow instability zone change significantly under different deformation conditions. With the increase in strain (from 0.1 to 0.7), the value of *η* declines (Figure 8). Moreover, it can be found that the peak values of the efficiency region are the strain rate range (0.01–0.1 s^−1^) and the temperature range (493–543 K) (Figure 8b,c).

From Figure 9a, the shaded regions increase rapidly at lower strain levels (0.1–0.3). Meanwhile, it can be seen that the shaded regions are mostly presented at low temperatures (423–473 K) and strain rates (0.1–10 s^−1^) (Figure 9b,c). To ensure excellent processability, the shaded regions should be avoided. According to the analysis based on the power dissipation map and the unstable map, the optimal processing region for the hot processing of Zn-2.0Cu-0.15Ti alloy is the temperature range (493–543 K) and the strain rate range (0.01–0.1 s^−1^).

### 3.5. Microstructure Evolution 

**Figure 10:**
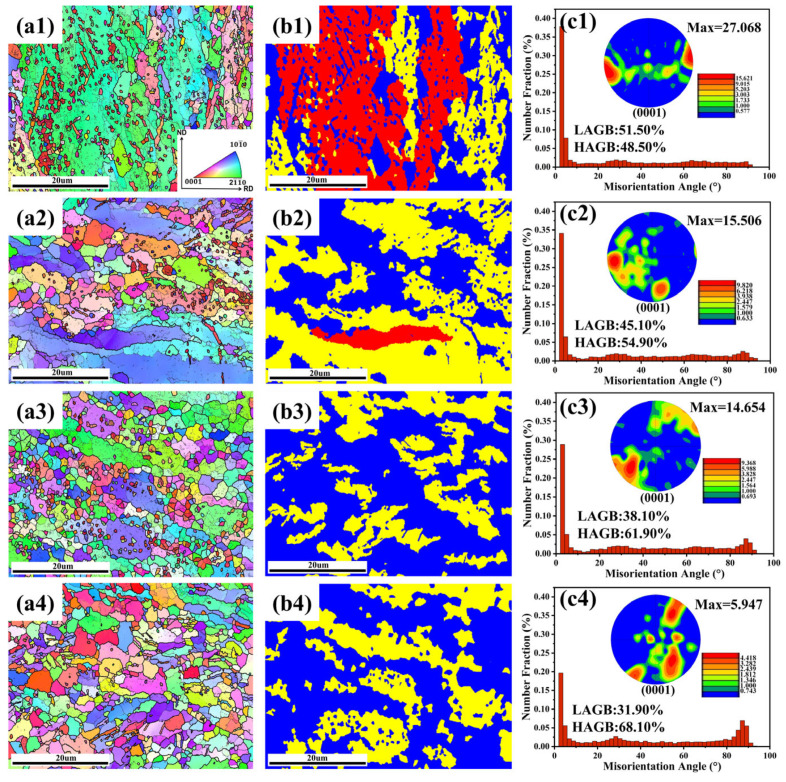
EBSD maps (**a1**–**a4**), recrystallization grain distribution maps (**b1**–**b4**) and misorientation angle (**c1**–**c4**) of the specimens deformed with strain rates of 1 s^−1^ at (**a1**–**c1**) 423 K, (**a2**–**c2**) 473 K, (**a3**–**c3**) 523 K, and (**a4**–**c4**) 573 K.

Figure 10(a1,a4) displays the EBSD maps of the specimens deformed at 1 s^−1^ and the deformation temperature of 423–573 K. In Figure 10(a1,a4), the low-angle grain boundaries (LAGBs, 2–15°) are described as fine black lines, while the high-angle grain boundaries (HAGBs, ≥15°) are described as thick black lines. It is dominated by elongated grains when the specimen deformed at 423 k (Figure 10(a1)). With increasing deformation temperature (from 423 to 473 K), elongated grains gradually disappear and are replaced by smaller rounded grains or equiaxed grains (Figure 10(a2)). As the deformation temperature rises, it shows that the DRX begins to occur. When T = 523 K, an increase in the amount of recrystallization fraction was observed, and some sub-grain boundaries from the original grains can be observed (Figure 10(a3)). Meanwhile, some fine grains develop along the original grain boundaries. The phenomenon is in line with the typical feature of discontinuous-type DRX (DDRX) induced by strain-induced boundary migration [33]. During deformation, the increase in HAGBs will hinder the continuity of dislocation slip [34] and cause stress concentration. In order to reduce stress concentration, the grain boundary bulges and migrates locally to form a distortion-free recrystallized nucleation. When T = 573 K, the recrystallized grains further grow, tending to equiaxed grain formation. The complete processing of the DRX is depicted in Figure 10(a4).

In Figure 10(b1,b4), blue denotes the fully recrystallized grains, yellow symbolizes the subgrains, and red represents the deformed microstructures. Figure 10(b1) shows that the microstructure underwent deformation with only a few subgrains and recrystallized grains. Low deformation temperatures cause inadequate recrystallization time. When T = 523 K (Figure 10(b3)), the deformed structure is replaced by the sub-grains and disappears. When the temperature exceeds 523 K (Figure 10(b4)), full recrystallized grains formed along the original grain boundaries can be detected. Therefore, the DRX volume percent is enhanced with increasing deformation temperature [34,35].

From Figure 10(c1,c4), it can be found that the proportion of LAGBs declines, and the proportion of HAGBs increases gradually as the deformation temperature increases [36,37]. The proportion of HAGBs increases from 48.5% to 68.1% when the deformation temperature rises from 423 to 573 K. This is because higher deformation temperature favors DRX, which causes subgrain boundaries to absorb dislocations and merge into large subgrains [38]. In addition, when T = 423 K, the maximum pole intensity of the basal texture in Figure 10(c1) is 27.068. However, when T = 573 K, the maximum pole intensity suddenly decreases to 5.947. The result indicates that the deformation temperature plays a significant role in the dynamic softening of the alloy [39].

**Figure 11:**
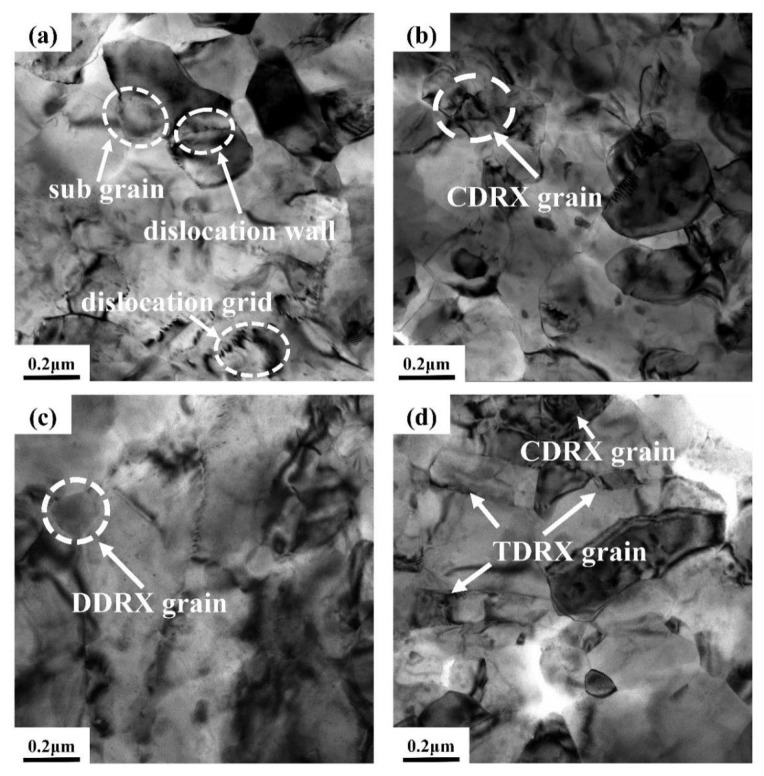
TEM micrographs of Zn-2.0Cu-0.15Ti alloy deformed at (**a**) 423 K/0.1 s^−1^; (**b**) 423 K/1 s^−1^; (**c**) 523 K/0.1s^−1^; (**d**) 523 K/10 s^−1^.

Figure 11 displays the TEM micrographs of Zn-2.0Cu-0.15Ti alloy under various deformation conditions. According to the results, the deformation temperature and strain rate are closely related to DRX [40,41]. Dislocation tangling and walls can be observed in the sample, along with original subgrains forming inside without obvious dynamic recrystallization grains in Figure 11a in the case of 423 K and 0.1 s^−1^. The softening of the alloy was mainly achieved through dislocation slip, cross-slip, climb, and recombination inside the grain [42]. When T = 423 K and $\dot{\varepsilon }$ = 1 s^−1^, a polygonal structure appeared clearly inside the grain due to the segmentation effect of dislocation grids or walls (Figure 11b), indicating the occurrence of continuous dynamic recrystallization (CDRX) inside the alloy. Within the strain rate range of 0.01–10 s^−1^, the elevated strain rate can enhance the effect of CDRX, due to the decline in the CRSSnon-basal/CRSSbasal ratio [12,43], which leads to CDRX occurring easily, weakening DDRX. At 523 K/0.1 s^−1^ (Figure 11c), an obvious curved grain boundary was observed on the original grain boundary. These bow-outs were the nucleation sites of discontinuous dynamic recrystallization (DDRX) [34,36]. Additionally, the elevated temperature increases dislocation mobility, which makes more dislocations tend to accumulate near grain boundaries (GBs). The sliding and moving rates of the GB are correspondingly increased due to higher driving forces at high temperatures. As a result, flow stress decreases (Figure 2c). However, as the strain rate increases to 10 s^−1^, twinning dynamic recrystallization (TDRX) and CDRX are observed (Figure 11d). This indicates that twinning is generated during the thermal deformation process, and twins can form much easier than slip at a high strain rate (10 s^−1^), due to the highly effective interface velocity [13] of the twin. A twin boundary (TB) will hinder the dislocation motion, thus increasing the strain energy around the twinning and providing the driving force for the nucleation of TDRX on TB. Meanwhile, the high strain rate increases deformation inhomogeneity, making dislocation slip more difficult, which leads to a significant increase in flow stress (Figure 2c) and the weakening of DDRX [13,44].

## 4. Conclusions

The hot deformation behavior of Zn-2.0Cu-0.15Ti alloy was investigated at strain rates 0.01, 0.1, 1, and 10 s^−1^ and deformation temperatures 423, 473, 523, and 573 K, using the Arrhenius model and 3D processing maps. The following are the primary conclusions:

1. The Arrhenius-type model is utilized to forecast flow stress behavior. The results show the Arrhenius model can accurately predict the flow stress behavior of Zn-2.0Cu-0.15Ti alloy.
2. Three-dimensional processing maps are generated at different strains based on DDM theory. The ideal processing domain for Zn-2.0Cu-0.15Ti alloy is the temperature range from 493 to 543 K and strain rate range from 0.01 to 0.1 s^−1^.
3. The softening mechanism of Zn-2.0Cu-0.15Ti alloy has diversification, including CDRX, DDRX, and TDRX, and is activated at T = 423–573 K and $\dot{\varepsilon }$ = 0.01–10 s^−1^. CDRX is activated at low deformation temperature (423 K) and high strain rate (1 s^−1^) and is inhibited with increasing deformation temperature and decreasing strain rate. However, when the deformation temperature increases (523 K) and the strain rate declines (0.1 s^−1^), DDRX becomes the primary softening mechanism, and is weakened with increasing strain rate. CDRX and TDRX become the main softening mechanisms when the strain rate is 10 s^−1^.

## Figures and Tables

**Table 1:** Chemical composition of Zn-2.0Cu-0.15Ti alloy.

Component	Cu	Ti	Impurity	Zn
Content (wt.%)	2.03	0.152	0.045	Bal.

**Table 2:** Coefficients of the polynomial equations.

a	n	Q	lnA
$h_{0}=0.0025$	$i_{0}=9.0261$	$j_{0}=25.071$	$k_{0}=14.882$
$h_{1}=0.1749$	$i_{1}=85.08$	$j_{1}=3023$	$k_{1}=454.91$
$h_{2}=-1.6323$	$i_{2}=-1147.2$	$j_{2}=-27,214$	$k_{2}=-4230.1$
$h_{3}=6.8839$	$i_{3}=5542.6$	$j_{3}=113,308$	$k_{3}=17,877$
$h_{4}=-14.666$	$i_{4}=-12,569$	$j_{4}=-238,415$	$k_{4}=-37,778$
$h_{5}=15.409$	$i_{5}=13,604$	$j_{5}=246,903$	$k_{5}=39,081$
$h_{6}=-6.3395$	$i_{6}=-5665$	$j_{6}=-99,939$	$k_{6}=-15,752$

## Data Availability

The data are available from the corresponding author upon reasonable request.
